# Systemic *Rasamsonia piperina* infection in a German shepherd cross dog

**DOI:** 10.1099/jmmcr.0.005125

**Published:** 2017-10-18

**Authors:** Joanna Lodzinska, Paola Cazzini, Claire S. Taylor, Jenifer Harris, Scott Kilpatrick, Tiziana Liuti, Gavin K. Paterson

**Affiliations:** ^1^​ Hospital for Small Animals, Royal (Dick) School of Veterinary Studies, University of Edinburgh, Roslin EH25 9RG, UK; ^2^​ Easter Bush Pathology, Royal (Dick) School of Veterinary Studies, University of Edinburgh, Easter Bush Campus, Roslin EH25 9RG, UK; ^3^​ Vets Now Referrals, 123–145 North Street, Glasgow G3 7DA, UK

**Keywords:** systemic *Rasamsonia piperina*, fungal osteomyelitis, lameness, pyrexia, voriconazole, caspofungin, amphotericin B, posaconazole

## Abstract

**Introduction.** Infection with the *Rasamsonia argillacea* species complex represents an emerging problem in human and veterinary medicine with systemic mycoses presenting with significant clinical complications and being a cause of death.

**Case presentation.** In this report, a case of systemic *Rasamsonia piperina* infection discovered in a 3-year-old male neutered, German shepherd cross dog is described together with the clinical presentation, the course of the disease and diagnosis. This report describes the first case of veterinary mycosis due to *R. piperina* in Europe and the first case in humans or animals in the UK.

**Conclusion.** Although seemingly rare, *R. argillacea* species complex infection should be a differential diagnosis for dogs, especially German shepherds with the described presenting signs, and radiographic and ultrasonographic findings.

## Abbreviations

ITS, internal transcribed spacer; RI, reference interval.

## Introduction

*Rasamsonia argillacea* species complex infections are an emerging problem in human medicine. In the absence of molecular diagnostics, morphological similarity to other fungi may cause misidentification of *Rasamsonia* species and thus their role as pathogens may be underappreciated. Veterinary *Rasamsonia* species infections have so far been limited to two previous cases and here we report a case of systemic *Rasamsonia* *piperina* infection in a 3-year-old, male neutered, German shepherd cross dog. In addition to being the first case of veterinary *R. piperina* mycosis in Europe and the first *R. piperina* case in humans or animals in the UK, this is also the first veterinary case of systemic mycosis due to *R. piperina* in a dog with macroscopic pulmonary, pleural, splenic, hepatic and lymphoid involvement.

## Case report

A 3-year-old, male neutered, German shepherd cross dog presented with a 4 week history of intermittent left hind limb, non-weight-bearing lameness. On physical examination the patient was resenting extension of hip joints and pain reaction was elicited on palpation of the left humerus. Initial treatment with meloxicam and tramadol did not resolve the clinical signs and the dog presented again 3 months later. On the second assessment the left hind limb lameness persisted and pain was also found on palpation of the left humeral, and both femoral diaphyses. Radiographs revealed a moderate, generalized and patchy increase in radiopacity in the medulla of the left femoral diaphysis. Smooth, continuous periosteal new bone formation extended along the medial and caudal cortex of the proximal third of the diaphysis. A diagnosis of left femoral panosteitis was made and the patient was discharged with exercise restriction and analgesia. The lameness failed to improve and the dog presented again a few days later, this time with shifting lameness involving left front and hind limbs and pyrexia. Recent lethargy, hyporexia, vomiting and restlessness were also reported.

**Fig. 1.**
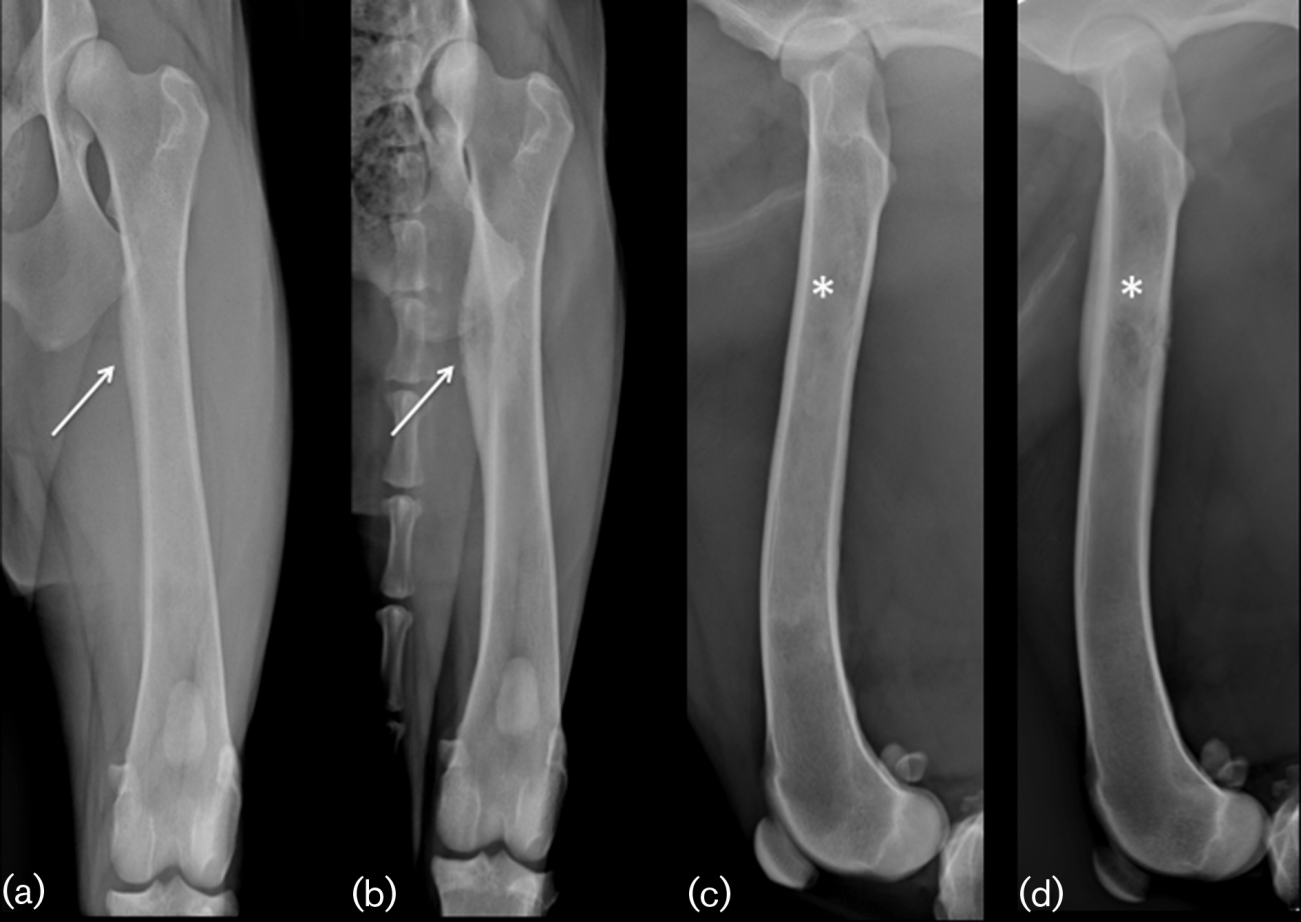
Caudocranial and mediolateral projections of the left femur on initial presentation (a, c) and on second assessment (b, d) showing disease progression. (a,c) Moderate, generalized and patchy increase in radiopacity within the medulla (star) and smooth, continuous periosteal new bone formation along the medial cortex (arrow). (b,d) Large region of mixed lysis and proliferation affecting the medulla of the proximal diaphysis (star) with associated marked lamellar periosteal reaction (arrow). Note moderate loss of muscle mass visible on the caudocranial view (b) when compared to the first assessment (a).

An investigation of pyrexia of unknown origin was initiated. Blood analysis revealed monocytosis [12.2×10^9^ l^–1^, reference interval (RI) 0–1.5], hypoalbuminaemia (21.5 g l^–1^, RI 26–35) and hyperglobulinaemia (42 g l^–1^, RI 18–37). Radiography of the right femur showed an aggressive proximal diaphyseal lesion, which had progressed compared to previous images, with secondary disuse muscle atrophy (Fig. 1). Differential diagnoses included fungal or bacterial osteomyelitis. An adjacent soft tissue neoplasm was considered less likely. Thoracic radiographs revealed partial right middle lung lobe consolidation with lobar pneumonia and small- to moderate-volume right-sided pleural effusion.

On abdominal ultrasound there was marked mass effect in the cranial abdomen associated with marked generalized lymphadenopathy, steatitis and minimal peritoneal effusion. The spleen was enlarged with irregular margins, and moderately heterogeneous. Thoracentesis was performed and fine needle aspirates were taken from the spleen, lymph nodes and mass in the cranial abdomen.

**Fig. 2.**
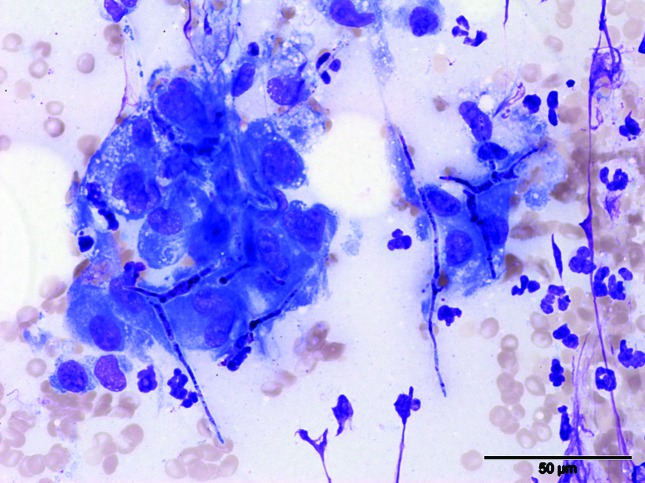
Image from the abdominal mass: note the presence of high numbers of macrophages associated with several fungal hyphae. A few neutrophils are also present in the background. Romanowsky staining, 400× magnification. Bar, 50 µm.

Cranial abdominal mass and lymph node fine needle aspirates were consistent with marked pyogranulomatous inflammation with multinucleated giant cells and intralesional fungal hyphae. Hyphae were 2–3 µm in width, and had parallel walls with septate and 45–90° branching. They stained purple, and had a think, clear outer cell wall (Fig. 2). The pleural effusion was consistent with eosinophilic to mixed inflammation, consistent with a reaction to the disseminated fungal disease. While waiting for culture results a working diagnosis of aspergillosis was made and treatment with itraconazole (5 mg kg^–1^ PO q24h), amphotericin B (1 mg kg^–1^ IV CRI) and terbinafine (20 mg kg^–1^ PO q12h) was initiated. The patient presented again the following day with an episode of bilateral epistaxis and worsening respiratory signs. Repeat radiographs showed moderate progression of pleural effusion and marked sublumbar lymphadenopathy associated with moderate peritoneal effusion, and hepatomegaly. The dog was persistently pyrexic, so itraconazole and terbinafine were withdrawn and treatment with voriconazole (5 mg kg^–1^ PO q12h) was commenced.

The patient was hospitalized and repeat abdominal ultrasound scans revealed new heterogeneous appearance of the liver with numerous hypoechoic lesions. Cytology of the hepatic lesions revealed marked pyogranulomatous inflammation with fungal hyphae and inter-hepatocellular bile casts, indicating cholestasis.

Microbiological examination of hepatic lesion fluid isolated a pure growth of a fungus described as having macro- and micro-morphological characteristics similar to those of *Purpureocillium* species and *Paecilomyces* species; no *Aspergillus* species was isolated. As the identity of the isolate was not clear from colony morphology or microscopic examination, molecular identification was used. An internal transcribed spacer (ITS) region of the ribosomal DNA gene cluster was amplified and sequenced on both strands with primers ITS-F and ITS-R [1]. The resultant 422 bp ITS region sequence was identified using the ISHAM-ITS database (http://its.mycologylab.org) [2]. The highest match was 99.763 % nucleotide identity to the ITS region of the type strain of *R. piperina* (CBS 408.73; NCBI accession no. NR_120176.1, position 71–492). To confirm the identity as *R. piperina*, fragments of the beta-tubulin and calmodulin genes were amplified and sequenced on both strands using the respective primer pairs Bt2a/Bt2b and Cmd5/Cmd6 as described previously [3]. Both sequences showed 100 % nucleotide identity with the respective genes of *R. piperina* CBS 408.73 (beta-tubulin JX273000.1, positions 45–439; and calmodulin JX272936.1, positions 43–422). Antifungal susceptibility was performed using the YS07 Vitek card (bioMérieux), and MICs were as follows: fluconazole 32 mg l^–1^, voriconazole 4 mg l^−1^, caspofungin 0.5 mg l^−1^, micafungin <0.006 mg l^−1^, amphotericin <0.25 mg l^−1^ and flucytosine <1 mg l^−1^.

## Outcome and follow-up

Despite the ongoing treatment the dog continued to deteriorate: large volumes of pleural fluid were continually drained and peripheral oedema developed. Due to the progressive decline, the owners elected to have the dog euthanized.

## Discussion

Infections with the *Rasamsonia* species complex are an emerging problem in human medicine, particularly in immunocompromised patients [4–8]. To date there have only been two previous case reports of *Rasamsonia* species infection in veterinary medicine [9, 10]. Both cases were of single German shepherd dogs infected with *R. argillacea* [10] and *R. piperina* [9] (initially reported as *Geosmithia piperina* [11]).

The main similarities in the two cases include age and breed. Systemic aspergillosis has been most commonly reported in German shepherd dogs between 2 and 8 years of age [12–16] and it has been suggested that a hereditary immune defect might exist [17–20] alongside mucosal immunity dysfunction [21]. This heredity susceptibility may extend to *Rasamsonia* species complex infection and highlights the need to consider other fungal causes beyond *Aspergillus* species for systemic mycoses in this breed.

Presenting signs in our case were similar to those of *R. argillacea* [10] where an initial lameness was the primary complaint. In the previous case report of *R. piperina* [9], the main presenting signs were acute-onset glaucoma of the right eye together with combined presence of lethargy, spinal hyperpathia, panuveitis, haematuria and pyuria. None of those were seen in our case, where the signs of systemic disease were limited to pyrexia and, later in the disease course, epistaxis and respiratory signs.

Both dogs in previous reports [9, 10] had radiographic signs consistent with discosponydlitis. In the case described here the initial radiographic findings were consistent with panosteitis, and lesions characteristic of osteomyelitis did not develop until later.

Neither of the previous veterinary *Rasamsonia* species mycosis reported macroscopic pulmonary or pleural involvement although that has been reported in human medicine in patients with cystic fibrosis undergoing lung transplants [4, 5, 8]. There were abnormalities on abdominal ultrasound of both previous dogs. The previously reported case [9] had bilateral renal pelvic dilation with all other organs appearing normal, unlike in our case where a clear splenic, hepatic and lymphoid involvement was present.

In the case described here the diagnosis of disseminated fungal infection was reached following fine needle aspirates from the spleen, lymph nodes and liver. Fungal hyphae were also observed in the pleural effusion following thoracocentesis, unlike the previous veterinary reports [9, 10] where the diagnosis was reached following aspirates or biopsies of the vertebral bodies or sternebra. Molecular characterization was pivotal in distinguishing the fungal species in all three reports.

Recent antifungal testing [11] on *Rasamsonia* species showed that caspofungin had significant activity *in vitro*, followed by amphotericin B and posaconazole. In another study voriconazole and isavuconazole had poor *in vitro* activity against *Rasamsonia* isolates and the echoinocandins were found to be the most effective agents [22]. Voriconazole was the least active of the antifungals tested [11, 22] and amphotericin B was added only late in the course of the disease, which may be the reason for the poor outcome in this case.

It is possible that previous cases reported as *Aspergillus* or *Paecilomyces* might have actually been *Rasamsonia* species infections. We agree with other authors [10] and suggest that molecular fungal identification is warranted in future cases, which so far has only rarely been done in veterinary publications. A study has successfully validated a commercially available real-time PCR assay that reliably detected three species from the *R. argillacea* complex direct from clinical specimens and from culture isolates [23]. We believe that the *Rasamsonia* species complex is potentially an underappreciated and misdiagnosed pathogen and that molecular approaches should be used in suspect cases because of the potentially aggressive disease pattern and refractoriness to medical treatment [6, 9, 11, 24].
